# An Exploration of the Factors Considered When Forming Expectations for Returning to Work following Sickness Absence Due to a Musculoskeletal Condition

**DOI:** 10.1371/journal.pone.0143330

**Published:** 2015-11-18

**Authors:** Amanda E. Young, YoonSun Choi, Elyssa Besen

**Affiliations:** Center for Disability Research, Liberty Mutual Research Institute for Safety, Hopkinton, Massachusetts, United States of America; Catholic University of Sacred Heart of Rome, ITALY

## Abstract

**Introduction:**

Workers’ own expectations for returning to work following a period of sickness absence have been found to be one of the best predictors of future work status; however, there is a limited understanding of why people expect what they do. The current study was undertaken with the aim of determining what people take into consideration when forming their expectations for returning to work.

**Methods:**

Thirty-four people (8 women, 26 men), who were off work due to a musculoskeletal condition, participated in one of 14 focus groups. Participants were aged 25 to 65 (M = 45, SD = 12.6), and all had been out of work for 3 months or less.

**Results:**

All participants reported expecting to return to work, with the most common timeframe being approximately 30 days (Range = 1 day-12 months). When explaining what they thought about when forming their expectations, participants referenced numerous considerations. Much of what was spoken about could be compartmentalized to reflect features of themselves, their condition, or their broader environmental contexts. Participant’s subjective experience of these features influenced his or her expectations. Prominent themes included concerns about employability, a desire to get back to normal, no job to go back to, mixed emotions, re-injury concerns, the judgments of workplace stakeholders, being needed by their employer, waiting for input, until the money runs out, and working out what was in their best interest.

**Conclusions:**

Indications are that many of the reported considerations are amenable to intervention, suggesting opportunities to assist workers in the process of returning to work.

## Introduction

As medical variables have been found to incompletely explain return-to-work outcomes, the role of psychosocial variables has become the focus of research attention. One such variable that has been found to predict future work status is the worker’s own expectations for returning to work [1]. While it has been suggested that individuals assess a myriad of physical, personal and environmental variables when forming their expectations for returning to work [2], there is little that is specifically known about the factors that people reference when asked to make predictions about their future work status. If we can understand the contributing factors, we will be better positioned to develop interventions that have the potential to remove perceived barriers and improve workers’ chances of achieving a timely, safe and sustained return to work.

Although there are no studies that have specifically asked about what people take into consideration when forming expectations for returning to work, past research provides some insight. In particular, prior research has addressed factors associated with return-to-work expectations, return-to-work uncertainty, as well as hesitations about, and potential obstacles to, returning to work. One such study was conducted with the aim of addressing the question: What influences positive expectations for return to work? The analysis of individuals with whiplash disorders indicated that depressive symptoms, lower education, lower income, male sex, and greater initial pain were associated with lower return-to-work expectations [3]. While this prior study provides some insight, those results do little to advance the understanding of the factors taken into consideration when formulating responses to questions about future work status.

The study by Stewart *et al*. [4] of workers’ construction of expectations for return to work in persons who were off work for 3–6 months with sub-acute back pain also provides insight. In that study, researchers found that their participants were reluctant to express their expectations because they were uncertain if they would be able to return to work and, if so, when and under what conditions. Rather than reporting on the factors being taken into consideration, the researchers reported that return-to-work expectations were constructed based on perceived uncertainty, which originates from five dimensions: 1) perceived lack of control of the return-to-work process, 2) a perceived lack of recognition by others of the impact of the injury, 3) perceived inability to perform pre-injury duties, 4) fear of re-injury, and 5) perceived lack of workplace accommodations. Although not directly focused on the formation of return-to-work expectations, another relevant study by Shaw and Huang, aimed to identify themes related to self-efficacy and expected outcomes associated with returning to work. Based on participants’ responses, the authors concluded that hesitation to return to work involved concerns about pain, re-injury, perceived ability to perform physical tasks, ability to meet role expectations, obtaining workplace support and maintaining job security [5].

Previous research on the formation of recovery expectations, which includes expectations for return to work as well as general recovery, may contribute towards our understanding of how return-to-work expectations are formed. Iles and colleagues [6] examined recovery expectations in individuals with non-chronic non-specific low back pain and found that the factors relating to the formation of recovery expectations included individuals’ pain levels, how their condition progressed, how pain impacted daily living functions, and their treatment experience. While the study sheds some light on the development of recovery expectations, it is possible that additional or different factors may be critical when focusing specifically on expectations for return to work.

Together with a lack of understanding of the factors people take into consideration when forming their return-to-work expectations, there is the absence of an understanding of whether influences differ depending on how long the person has been off work. Within the return-to-work literature, there is research suggesting that work disability is developmental in nature. A model that has received empirical support is a 3-phase model of work disability in people with low back pain [7]. This model distinguishes phases defined by the number of days off work: acute (up to 1 month), sub-acute (2–3 months), and chronic (more than 3 months) [7]. From a clinical perspective, those out of work for three months or more (i.e. those in the chronic phase of disability) tend to represent complex cases who require intensive, multidisciplinary interventions to achieve improved return-to-work outcomes [8]. When targeting a population for research purposes, with the eventual aim of improving outcomes, it would seem optimal to include people who had been out of work long enough to formulate ideas about if and when they will return to work, yet still be at an early enough stage where targeted interventions based on specific factors would be most useful. As such, in the current study, we chose to focus on workers in the acute and sub-acute phases of work disability.

To summarize, while injured workers’ return-to-work expectations have been found to be one of the best predictors of their return-to-work outcome, little is known about what people consider when forming their expectations. Also lacking is an understanding of whether influences vary depending on how long the worker has been off work. To address this knowledge gap, the current study aims to gain an understanding of what people who are in the early phases of work disability take into consideration when forming their expectations for returning to work.

## Method

### Study design

The method chosen to address our research question was qualitative description [9, 10]. Qualitative methodologies have been found to offer a valuable depth of understanding of the perceptions and experiences of pain suffers [11]. Focus groups were chosen as a mechanism for data collection because it was felt that a conversation that involved others in a similar situation would result in expression and storytelling. In addition, focus groups allow the researcher to hear participants’ metaphors for their problems and gain an understanding of the contextual relationships [12]. A purposive sampling strategy was used. In qualitative research, the application of simple statistics, such as cross-tabulations, provides a means of identifying and confirming regularities and variations within the data (Dey, 2003). We chose to use this technique based on our desire to identify and elaborate the more prevalent considerations.

### Participants

The study inclusion criteria stipulated that participants had to be of working age, working at least 35 hours per week at the time of injury and currently off work due to a musculoskeletal condition, but for no longer than three months. Of the 145 people who contacted us indicating an interest in participating in the study, 77 persons did not fulfill the inclusion criteria. In the majority of cases (n = 45), this was because they had been off work for too long. Other reasons included not experiencing a musculoskeletal injury (n = 11), already being back at work (n = 4), and working less than 35 hours per week at the time of their injury (n = 4). Of those who did fulfil the inclusion criteria (68 persons), 34 participated in the research and 8 were scheduled to participate but did not attend. The remaining 26 were not sampled because it was felt that saturation had been achieved. Participants were selected based on their availability to attend the scheduled focus group sessions (morning, afternoon, and evening timeslots were offered).

In total, 34 people (8 women, 26 men) were sampled. Participants’ ages ranged from 25 to 65 years old (*M* = 45, *SD* = 12.6). People were off work for a variety of conditions including back pain (n = 15, 44%), upper limb injury (n = 10, 29%), lower limb injury (n = 8, 24%) and chronic pain (n = 1, 3%). All had been out of work for 3 months or less due to their work-disabling musculoskeletal condition (*M* = 46 days, Range = 18–86 days). Most had a work-related injury (n = 25, 74%); however, only 10 (29%) had filed a Workers’ Compensation claim. Most participants (62%) held physically demanding jobs. Discussions revealed that the participants’ level of educational achievement varied widely, as did their relationship status, the presence of dependents, and their career aspirations. At the time of participating in the focus groups, all participants reported expecting to return to work. Most commonly, participants expected to return to work in approximately 30 days from the date of the focus group (*M* = 47 days, Range = 1 day-6 months). See Table 1 for more details regarding participant characteristics and predicted time frame for returning to work.

**Table 1:** Participant (N = 34) characteristics and expected timeframe for returning to work.

IDǂ	Gender	Age	Occupation	Injury	Work-Related	WC Claim Filed	Job to Return to	Estimated Time to RTW
FG1.1	M	57	Office worker	Hip injury	Yes	Yes	Yes	6 weeks
FG1.2	M	35	Sales professional	Knee injury	Yes	Yes	Yes	10 days
FG2.1	M	63	Maintenance	Shoulder injury	Yes	Yes	No	6–12 weeks
FG2.2	M	65	Driver	Back pain	Yes	Yes	Yes	1 month
FG3.1	F	49	Patient care assistant	Back pain	Yes	No	Yes	2 weeks
FG3.2	M	49	Landscaper*	Back pain	Yes	No	No	2–3 weeks
FG3.3	M	47	Carpenter	Fractured toes	Yes	Yes	Yes	1 week
FG4.1	M	53	Landscaper*	Torn bicep	Unsure	No	No	2 weeks
FG4.2	M	43	Office worker	Back pain	No	No	Yes	2 weeks
FG4.3	F	50	Patient care assistant	Back pain	No	No	Yes	12–19 days
FG4.4	M	27	Mechanic	Fractured elbow	No	No	Yes	6 days
FG5.1	F	56	Clinician*	Leg pain	Yes	No	Yes	3–12 months
FG5.2	F	36	Restaurant service	Back pain	Yes	No	Yes	11 days
FG5.3	M	49	Studio assistant	Back pain	Yes	No	Yes	15 days
FG6.1	M	29	Construction worker	Back pain	Yes	No	No	3–9 months
FG6.2	M	52	Shop assistant	Back pain	No	No	Yes	2 weeks
FG6.3	M	34	Restaurant service	Hand injury	Yes	No	Yes	1 day
FG7.1	F	34	Office worker	Chronic pain	No	No	No	3–4 weeks
FG8.1	M	29	Landscaper	Knee injury	Yes	Yes	Yes	6 months
FG9.1	M	24	Office worker	Wrist pain	Yes	No	No	3–6 months
FG9.2	M	28	Landscaper	Back strain	Yes	No	Yes	1–2 weeks
FG9.3	F	54	Marketing professional*	Back pain	Unsure	No	Yes	1 week
FG10.1	M	25	Mechanic	Back pain	Yes	No	Yes	3 months
FG10.2	F	60	Office worker	Hand pain	No	No	Yes	4–5 weeks
FG10.3	M	31	Construction worker	Back pain	Yes	No	No	3 months
FG10.4	M	63	Construction worker	Shoulder injury	Yes	Yes	Yes	10 weeks
FG10.5	M	50	Office worker	Shoulder injury	Yes	No	Yes	2–3 weeks
FG11.1	M	50	Nurse aide	Fractured elbow	No	No	No	3 months
FG11.2	F	55	Patient care assistant	Leg pain, Knee pain	Yes	No	No	3 months
FG11.3	M	35	Massage therapist	Hand sprain	Yes	Yes	Yes	10 days
FG12.1	M	52	Office worker	Knee sprain	Yes	No	Yes	2–6 weeks
FG13.1	M	55	Driver	Back pain	Yes	Yes	Yes	1 month
FG13.2	M	59	Sales professional	Back pain	Yes	Yes	Yes	4 weeks
FG14.1	M	30	Teacher	Foot pain	Yes	No	Yes	2 weeks

ǂ Identification code indicating the focus group in which the individual participated, and his or her number within the group (e.g. FG1.1 = Focus Group 1, Participant 1).

* Self-employed. RTW = Return to work.

### Procedure

Participants were recruited through advertisements in local newspapers and digital media (Craigslist, Monster.com and local online newspaper). No efforts were made to compose groups based on socio-demographic characteristics. The groups were facilitated by three researchers experienced in conducting qualitative research; all were female and had backgrounds that included the conduct of research into factors influencing return to work. Group facilitators were unknown to the study participants. Focus groups were held between February and August, 2013 at our research facility located in the greater Boston area, Massachusetts, USA. Although all groups were scheduled to include two facilitators and a minimum of three participants, in three instances “no shows” resulted in only one participant being present. Rather than rescheduling, the two facilitators interviewed the sole attendee. For ease of reporting, focus groups and two-on-one interviews are collectively referred to as “groups”.

Prior to the commencement of the focus group, each participant completed the written informed consent process. Anonymity and confidentiality issues were discussed and participants were insured anonymity in any presentation or publication of the study findings. Participants were also told about the aims of the research and the reason it was being conducted. All procedures followed were in accordance with the Helsinki Declaration of 1975, as revised in 2000, and the study was approved by the New England Institutional Review Board.

Once consent had been received, each participant completed a questionnaire inquiring about: (i) whether they expected to return to work (yes/no), (ii) if so, the approximate timeframe for doing so (asked to specify number of days/weeks/months until expected return to work), and (iii) the factors they considered when forming their responses. Participants were instructed to list as many influences as they wanted to. A full lined page was provided. Typically this task took 5 to 10 minutes to complete. Once completed questionnaires were collected, participants were then asked to briefly introduce themselves. The lead facilitator then went through the influences listed by the participants, asking the relevant participants to expand on their responses, and then asking others to share similar or contrasting experiences. Throughout the discussions, the lead facilitator checked in with participants regarding her understanding of what was being discussed. This involved the researcher summarizing what people had been talking about and asking the group if the summary was accurate. Each data collection session was approximately two hours in duration.

After the completion of each group, the facilitators met to debrief and code observations. A record was kept of ‘newly’ reported considerations. Sampling continued until the facilitators felt as though they were not accessing new information (i.e. saturation had been reached). Focus groups were audio recorded and recordings were fully transcribed (verbatim) by an experienced transcriber. Transcripts were then checked for accuracy. Corrections were made in cases where words where misheard by the transcriber.

### Analysis and data presentation

Transcripts were subjected to summative content analysis, which involved the counting of reported considerations, followed by the interpretation of the latent context [13]. Consistent with the qualitative description approach [9, 10], response codes were counted; but rather than counts being an end, counts were used to identify patterns and regularities in the data. There were several steps to our analytical process. First, we reviewed the transcripts to gain a sense of emerging themes. Then, we began the process of coding the focus group transcripts. Rather than assigning *a priori* codes, two researchers independently reviewed the data and identified thematic content from within the data.

The process of code generation revealed that participants referenced the same factor in different ways. For example, participants commonly spoke about recovery; however, some wanted a full recovery before returning to work, while others were willing to return to work before being fully healed. As our process evolved, we found that: 1) much of what people spoke about could be compartmentalized to reflect features of themselves, their condition, or their broader environmental contexts, and 2) that the participant’s subjective experience of these features influenced his or her expectations. To capture this, we adopted a double-coding strategy such that participant comments were coded as having an “element” and a “lived experience” component. Elements were defined as features of the individual, his or her work-disabling condition, and the environmental contexts (work disability management, work, social, physical, and economic) within which he or she operated. Lived experiences were defined as the worker’s personal interpretation of, interaction with, or evaluation of the relevant element. Put another way, an element can be thought of as the referent object, and the lived experience is the personal meaning that the individual applies to that object.

By the end of the analytical process, we settled on seven element and nine lived-experience groupings. Lived experiences were divided into two categories: those that occurred internally (as within or under the control of the participants) and those that involved input from the external world. Within-the-individual codes were grouped to reflect participants’ behaviors, wants, feelings, beliefs, and tasks that needed to be worked through. Behaviors were things people physically did that influenced their expectations. Wants encompassed participants’ reported needs and desires. Feelings encompassed reported internal emotional states. Beliefs reflected participants’ perceptions of the world around them. Tasks were generally cognitive in nature, and involved decision making or processes that needed to be worked through. Lived experiences involving input from broader contexts were categorized into four groups reflecting: access to resources, relationships and/or interactions with others, the occurrence of an event, and the passing of time. Further information regarding the number and examples of codes contained within these groupings can be found in Table 2. Once the element and lived-experience codes had been finalized, the questionnaire responses and transcripts were again reviewed and, where possible, lived-experience codes were applied to referenced elements.

**Table 2:** Coding structure displaying code groupings, number of codes going into each grouping, and examples of codes falling into each of the groupings.

Code-Groupings			No. of Codes in Grouping	Examples of Codes
Elements				
	Self		29	Employment status, Financial position, Prior injuries, Work routine, Stage of life
	Work context		25	Employer, Income, Job to go back to, Supervisor, Working conditions
	Work-disabling condition		14	Pain, Healing, Recovery, Re-injury, Work-relatedness, Compensated
	Disability-management context		10	Doctor, Therapists, Treatment, Medication, Surgery, Diagnosis
	Social context		6	Family, Peers, Social networks, Role models, Society
	Economic context		3	Economy, Welfare, Litigation
	Physical context		2	Season, Distance
Lived Experiences				
	Within the individual			
		Beliefs & Perceptions	20	Everything will work out, They don’t care, Holding me back, Difficulties with
		Something to be worked through	18	Looking for, Considering, Working out what is best for me,
		Wants & needs	11	Want to regain, Want to maintain, Want to progress, Want to contribute
		Behaviors	10	Doing, Avoiding, Staying connected, Job search activities
		Feelings	7	Concerned, Enjoys/likes, Uncertain, Depressed about, Fear of losing, Fear of becoming
	Involving input from broader context			
		The occurrence of an event	8	Clearance from, Depends on, Expiration of, Outcome of, Will return when
		Access to resources	7	Availability of, Presence of, Absence of, Limited in, Opportunity to, Loss of
		Interactions with others	5	Relationship with, Social support to/from, Economic support to/from
		The passing of time	1	A timeframe

In this paper, we chose to focus on considerations that were most commonly referenced. To identify these, we cross-tabulated element codes against lived-experiences codes using NVivo 10 (QSR International). More highly populated element-by-lived-experience intersections (e.g. recovery by concerns) were examined with the aim of identifying prominent themes. This involved two researchers reviewing the text within a selected intersection for manifest and latent content (i.e. the underlying meaning). The analytical process was iterative and involved generating codes, checking for redundancy, and reviewing the coding application to confirm thematic similarity. To address reliability [14], the researchers met throughout the analytical process to discuss coding revision and application. If disagreement occurred, this was resolved through re-examination of the data and discussion until agreement was achieved. Once the coding structure had been developed, this was reviewed by the third author for credibility and resonance [15]. In addition, the third author assisted with the identification of prominent themes.

When presenting findings, we begin with describing the elements and lived experiences separately. Next, we describe the intersections between the elements and lived experiences. Finally, we explore prominent themes within the elements by lived-experiences intersections. When interpreting findings, it should be noted that numbers should not be interpreted as concrete–they are a means, not an end. It is highly likely that there were instances where codes were not applied where they could have been. Rather than focus on the absolute number, the reader should view the frequency of references as an aid to gaining a sense of the prevalence of the consideration. So as to ground our interpretations [15], representative quotes were chosen to reflect observed themes. [Note: ID codes (e.g. FG1.1) are provided following quotes. These can be referenced in Table 1 to obtain the relevant participant’s demographic and injury information. In some cases, quotes were edited to improve readability.]

## Results

### Elements

In total, we identified seven element groupings: elements of the self, the work-disabling condition, and the disability management, work, social, physical, and economic contexts. For details regarding how frequently these were referenced, see Table 3. The most frequently mentioned element referenced characteristics of the self. These included: age, skills and abilities, financial position, and their sense of self. While frequently mentioned, age tended to be described less as a number, and more in terms of where they were in their stage of life, with references being made to family, lifestyle and approaches to work. Abilities included skills, as well as functional capacity related to general health, their current work-disabling condition, and/or prior injuries. Sense-of-self includ**e**d pride, identity as a worker, along with being productive and honest. Elements of the work context could be split into whether or not the participant had a job to return to. For those who did have a job to go back to (n = 24), participants mentioned features of the job that could influence the ease with which they could return (e.g. flexibility in adjusting their work schedules in terms of hours and/or duties, the physical and mental demands, and stress). For those without a job to go back to, the “right job” was often spoken about. Elements of the participants’ work-disabling condition that were most commonly discussed included: type of injury, healing/recovery, and the potential for re-injury or chronicity. Other important aspects were the work-relatedness of their condition and whether or not their time off work was compensated. Commonly mentioned elements of the disability-management context included components of participants’ healthcare, such as getting the diagnosis and treatment (e.g., medication(s), surgery, or therapy) as well as the influence of their doctor and physical/occupational therapist. Less frequently mentioned elements referenced social, economic and physical contexts. Elements of the participants’ social context included their social networks, such as their families, friends and peers. More commonly discussed elements of the economic context were the economy, work opportunities, welfare payments and litigation. Features of the participant’s physical contexts (e.g., weather, commute) were mentioned, but with relative infrequency.

**Table 3:** Coded verbal responses to our request for participants to further explain the things they took into consideration when responding to our asking about their expectations for return to work.

		Elements							
	Lived Experiences	Self	Work-disabling condition	Disability-management context	Work context	Social context	Physical context	Economic context	All
		Refs	Refs	Refs	Refs	Refs	Refs	Refs	Refs
Within the individual									
	My behaviors	7	5	16	15	0	0	0	41
	My wants	89	21	5	30	10	2	1	126
	My feelings	95	58	18	68	10	3	7	193
	My beliefs	39	32	24	58	6	3	6	127
	Tasks I need to work through	44	21	31	43	2	0	7	107
Involving input from broader context									
	My access to resources	47	14	8	72	1	3	8	123
	My interactions with others	17	4	1	55	12	0	2	73
	The occurrence of an event	19	23	51	12	2	1	4	88
	The passing of time	16	20	17	4	0	0	1	44
Total		295	155	134	272	32	10	26	729

Note: Figures represent the number of “considerations” coded to include the relevant “element” and “lived experience.” Each reference (Ref) denotes an instance where the study participant mentioned the relevant element and lived experience when talking about what they took into consideration when forming their expectations for returning to work. A consideration may have been coded to include more than one element and more than one lived experience. As such, lived-experience and element totals do not necessary reflect a summation of the groupings.

### Lived experiences

A summary of participants’ lived experiences is also contained within Table 3. The most frequently mentioned lived experiences occurred within the individual. Feelings and wants were most prominent. Participants described feeling bored, depressed, frustrated, stressed, and uncertain. Examples of wants included wanting to contribute, to be treated fairly, and to “do the right thing.” Less frequently spoken about was the impact of beliefs; however, these definitely exerted a strong influence. Beliefs tended towards a negative tone: “they don’t care,” and quite common was the perception that someone or something was “holding me back,” had “changed the rules,” or was sending “mixed messages.” However, there were also positive beliefs that appeared to facilitate the expectation of a timely return, including: being needed, perceptions regarding loyalty and readiness. Tasks that participants talked about having to work through included: considering options, making choices, and waiting for input. Behaviors typically involved avoiding or limiting their activities, and actions performed as preparations for return to work. The most prominent lived experience involving input from external contexts was access to resources and included both the absence and presence of said resource. The occurrence of an event and interactions with others were also commonly spoken about. The occurrence of an event typically involved the following: obtaining clearance, dependent upon some interim outcome, and receiving input from an advisor or decision maker. Interactions with others included relationships with employers, co-workers, peers, family and friends. These were described as delaying or encouraging a return to work, and involved both emotional and economic support. Timeframes were not as frequently mentioned; however, there were cases where participants reported needing to wait for a period of time before they could return.

### Intersections and prominent themes

**Fig 1:**
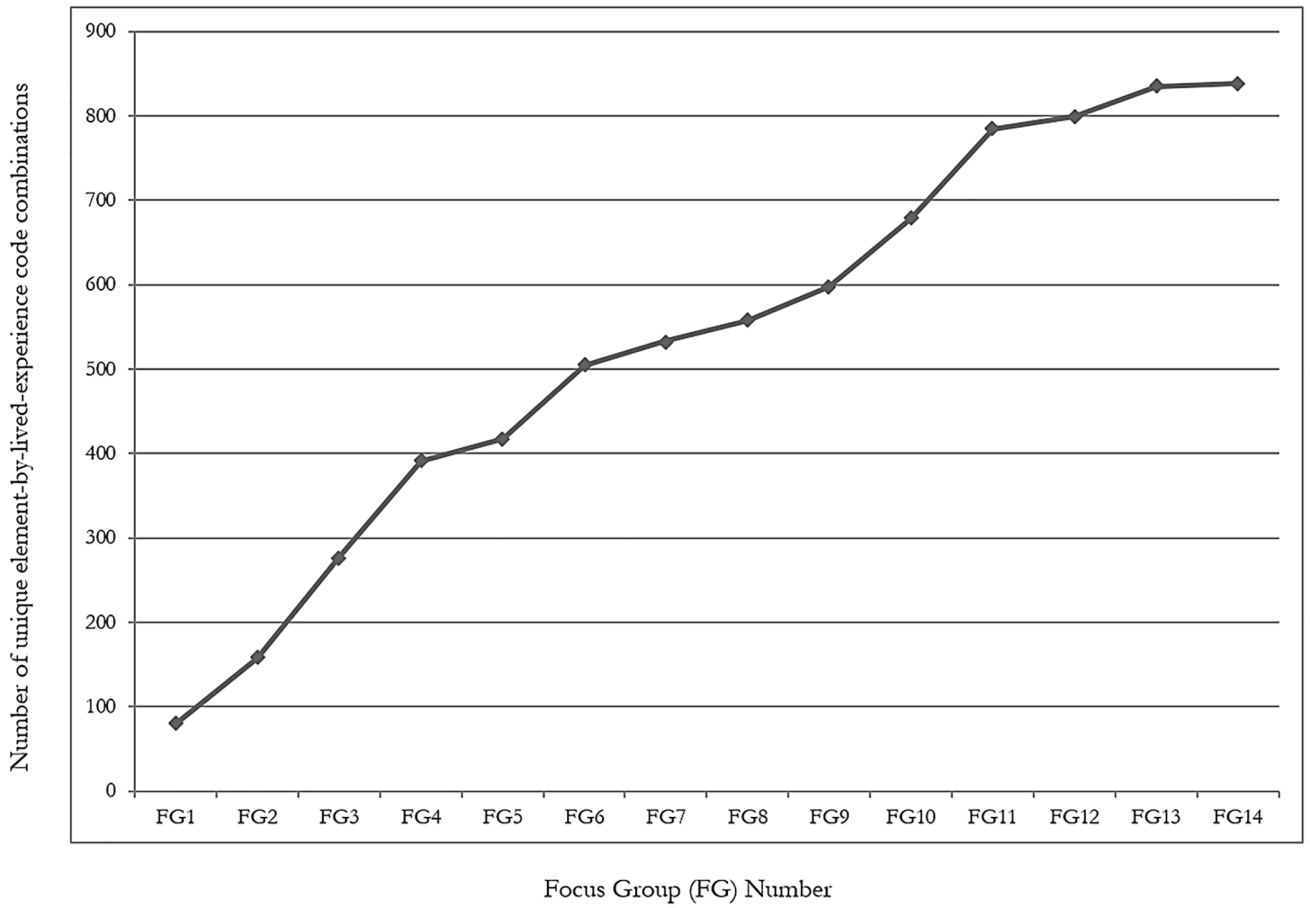
Number of unique element-by-lived-experience coding combinations occurring over the data collection period.

We observed 838 unique element-by-lived-experience code combinations (intersections). An analysis of the frequency with which new combinations were observed was performed to assess saturation. Inspection of Fig 1 reveals that the rate at which new observations occurred plateaued at around focus group 11. Only three new coding combinations emerged as a result of the conduct of focus group 14. When element-by-lived-experience groupings were compared, 56 unique intersections were observed. Analysis of the more frequently observed intersections (see Table 3) allowed us to derive 10 prominent themes. These included: concerns about employability, a desire to get back to normal, no job to go back to, mixed emotions, re-injury concerns, the judgments of workplace stakeholders, being needed by their employer, waiting for input, until the money runs out, and working out what was in their best interest. These are elaborated below.

The main theme to arise out of the self-by-feelings intersection was “concerns about employability.” Within this intersection, many participants, especially the older workers, expressed concerns about having limited opportunities for employment. Some also had concerns about being easily replaced. Typically, the sentiment was along the lines of: I need to get back to work, or no one is going to want to hire me.

I’m not foolish. I’m 63 years of age. […] THAT, more than anything else is my biggest question mark because of my age and my injury history. People are gonna be reluctant to hire me. (FG2.1)

Within the self-by-wants intersection, the main theme to arise was a “desire to get back to normal.” Participants expressed that working was a part of their self-identity and they wanted to go back to work to regain their sense of pride and accomplishment. There were also participants who felt that they were being perceived negatively for not working; as such, they wanted to return to regain their social standing. The typical sentiment was: the sooner I can get back, the better.

I’ve always worked my entire life and anytime I have to be home, it’s really not good for me or the others there. I mean, I do “house stuff” and “mother stuff” but, I like to fit the “house stuff” and my “mother stuff” in between work, as opposed to me, just like… It’s just not good for me. It’s not me, you know? You want to feel like you’re contributing. […] you want to contribute and feel like you’re part of something. (FG9.3)

For the work-context-by-access-to-resources intersection, the main theme we observed was having “no job to go back to.” In general, this intersection reflected the absence or presence of work opportunities. A number of participants indicated that were not able to go back to their pre-injury job, but that they were looking for other work. This was particularly difficult for a number of the self-employed participants who had lost clientele due to their work-disabling conditions. Typically, the sentiment was: It’s hard to say how long it will take, that depends on how long it takes me to find work.

Being self-employed, though, like I am at the moment? I lost customers […] I’ve had two good customers call me up and say: “we need you here now.” And I said, “I’m sorry, I can’t make it.” […] I’m not answering to one supervisor/boss, etc., I’m answering to a NUMBER, and I’ve lost two good accounts, now. They’ve gotten somebody else. But, four to six weeks, some people can’t wait. […] It’s upsetting but … you just, what can you do? (FG4.1)

A prominent theme observed within the intersection of work-context-by-feelings was having “mixed feelings.” Feelings as they related to the work context were varied and complex. They were spoken about in terms of being bored not working and missing their regular routine, but there were also concerns about what might happen when they returned. The typical sentiment was: I miss my work, I am bored at home, but I am concerned about what might happen if I go back too early.

So, the past couple of years, I’ve been teaching all subjects, sort of daunting … but, you know, I loved it. […] So, it’s kind of hard, sitting at home. You know (chuckles), when I could be working with them [my students] and having a great day. BUT… at my age, I’m thirty-years old, I don’t want to injure myself to the point, where … I’ve seen people in my family not be able to go to work because of injuries. And I don’t want to be like that as I get older. (FG14.1)

The most prominent theme arising out of the disabling-condition-by-feelings intersection was “re-injury concerns.” This typically involved concerns about going back to a situation that had resulted in them hurting themselves. Typically, the sentiment was: I want to make sure it’s safe; I don’t want to hurt myself, exacerbate my condition or have it become chronic.

One of my fears is … (sigh) … going back into the same thing I am coming out of … there’s … that fear of going back and re-hurting myself or hurting myself worse. I don’t want this to be a gray cloud over my head. (FG4.3)

A common theme within the intersection between work-context and interactions-with-others was the “judgments of workplace stakeholders.” In this intersection, relationships with employers/supervisors and co-workers were prominent. People spoke about wanting to be able do their job well and not being seen as a liability. Typically, the sentiment was: I want to go back, and others to see I can do my job.

I wouldn’t want people to look at me like I’m a liability. Or anything like that. […] I don’t want my employer to think I … “Well, geez, you know, everything we’ve done for him. Why would he be, uh … you know, be pulling this?” I don’t want them to think that, so I’m in constant touch with my supervisor, you know, MY direct supervisor. (FG1.1)

A theme that emerged from the work-context-by-beliefs intersection was being “needed by employer.” The belief that they were needed by their employer, mixed with feelings of loyalty was associated with feelings of urgency for return; however, this could also be perceived as a stressor, which had the potential to cause hesitation for people still recovering from their aliments. Typically, the sentiment was: My employer needs me to go back, but I am not sure I am quite ready.

I know everything that needs to be done. He couldn’t hire someone else to do all the stuff I do, because I know his patterns, and you know … he’ll get cranky. […] I feel guilty not helping my boss. [But] there’s like stress, and I have a lot of anxiety […] especially when I see [my employer] and his girlfriend arguing … that DEFINITELY stresses me out. I just can’t deal with that kind of anxiety in the workplace. (FG5.3)

For the disability-management-by-occurrence-of-an-event intersection, “waiting for input” was a core theme. This intersection tended to reflect waiting for clearance for return to work from a medical provider. In most cases, it was pretty straight forward; however, there was a case where clearance had been given by one doctor, but the opinion of a second provider had been requested. In the participant’s opinion, this was to protect the employer from litigation (FG4.2). Beyond getting clearance from a provider, there were those who needed to get the “all clear” from their employers. And there were others still waiting for a decision on their Workmen’s Compensation claim before they could seek medical treatment. Typically, the sentiment was: Waiting is delaying the process.

So, pretty much the past couple of weeks—it hasn’t just been a matter of healing; it’s waiting in limbo, to even go BACK to start the whole rehabilitation process. […] I can’t even think of, like, anything other than the obvious. I’m honestly just waiting for the comp claim to be processed so I can finally do the rehab work. So I can actually go to the doctor’s again. As soon as he signs off, I’m back! (FG8.1)

A theme to arise from the self-by-access-to-resources intersection was “until the money runs out.” This intersection typically reflected the participants’ financial position, as in the availability of financial resources to see them through their convalescence. In the following example, the participant was thinking about going back to work before his doctor scheduled a return. Typically, the sentiment was: At some point I am going to go back regardless of my condition; I have bills to pay.

We are starting to … you know … feel a financial pinch. I might even try to … you know … expedite my return. (FG12.1)

Within the self-by-something-I-need-to-work-through intersection, the theme was an awareness that they should take the opportunity to “work out what is best for me in the long term.” Participants reported wanting to take their time, do their research and make good decisions. More specifically, they spoke about needing to figure out if they needed to find a job that would be less likely to result in injury / aggravate their condition. They also reported needing to consider what they could do. Age was again something that played an important part in this intersection. Typically, the sentiment was: I want to work out what is best for me in the long-term. I know it’s working, but I need to think it through and get the right job for me.

[This time] the doctors looked at me differently than they did when I was in my 50s. [Now] they were looking at me as an older patient. They said “have you considered disability?” and that was never given to me before, because they assumed that, when you are younger, I guess, you are going to go back to work. When you get to be older, you’ve got to think where you are going and what you want to do. [I decided] I would like to work a few more years because I will make more money, and I actually like to work anyway. So I think that’s a big part of my consideration too. (FG13.2)

## Discussion

Study results indicate that there are many factors taken into consideration when forming expectations for returning to work. While expectations for recovery and the input from healthcare providers were important, these were only parts of the equation. The elements that participants reported covered a variety of health, functional, personal, and contextual variables. Beyond recovery expectations, which were recently found to be predominantly pain, condition progression, performance limitations and impact of treatment [6], the current study participants had numerous additional considerations, such as having a job to return to, wanting to get back to the normal routine, and feeling like they were needed at their jobs. Many of these considerations were in line with previous research into factors related to return to work. For example, past research has found that attitudes and beliefs [16, 17], as well as social interactions [18–20] relate to return to work. In the current study, these were all referenced as influencing expectations. Similarly, aspects of the work context, which we found to be a salient part of the formation of return-to-work expectations, have been found to consistently relate to return to work [21, 22] In contrast to earlier works [5, 23], in which participants indicated that pain was a consideration, this was not one of our major themes.

Similar to previous research showing that motivation to return to work is related to long-term employment outcomes after being off work due to musculoskeletal disorders [24], our findings suggest that wanting to get back to work, and back to the normal routine, plays a prominent role in the formation of return-to-work expectations. The observation that waiting for input, or clearance from others influenced return-to-work expectations is also consistent with earlier research. For example, Stewart *et al*. [4] reported that a perceived lack of control of the return-to-work process produced uncertainty, which contributed to individuals’ reluctance to form expectations for returning to work. Findings also suggest that a delayed return to work may be associated with uncertainty relating to concerns about what might happen when the worker goes back. In particular, concerns about performance and the judgment of others fall into this category. Several participants expressed re-injury concerns, and indicated that this influenced their expectations because they didn’t want to risk going back until it was safe. This is also in line with previous work suggesting that workers’ perception of being able to perform their work without being reinjured is an important factor influencing return to work [5, 23]. Findings suggest that there are factors that influence individuals’ expectations for returning to work that result in their expecting to return before being physically and/or mentally ready. Participants mentioned apprehensions about losing their jobs and being unable to find new employment because of their age and/or previous injury history. This is consistent with previous research illustrating the importance of monetary concerns on return to work [25]. However, it is somewhat inconsistent with an earlier work indicating that concerns about maintaining job security was associated with a hesitation to return to work [5].

Given that our results indicate that factors similar to those that relate to return-to-work outcomes are considered in the formation of return-to-work expectations, it is possible that return-to-work expectations may act as an intermediate variable in the relationship between those factors and return-to-work outcomes. It may be that these factors are exerting their influence on return-to-work outcomes by shaping individuals’ expectations for return to work which, in turn, results in behaviors that facilitate or hinder actually returning to work. There is some support for this possibility in previous research that found that return-to-work expectations mediated the relationship between return to work and fear avoidance-beliefs, organizational support, and return-to-work confidence [26]. Future research is needed to test this possibility.

The current analytical strategy was emergent, meaning that predefined codes were not applied; however, findings suggest that data could be fitted into models of work disability. For example, as was done in a recent study of vocational rehabilitation from the client’s perspectives [27], the structure afforded by International Classification of Disability and Health (ICF) could have accommodated the elements identified. While this exercise was considered, we chose not to pursue this path as we felt that doing so dulled the richness of the data with regards to the participants’ lived experience.

### Implications for practice

Study findings have several implications for practice and highlight opportunities for intervention. Findings suggest that addressing participants’ beliefs and perceptions could be associated with improved outcomes. Many of the quoted beliefs and perceptions were negative in nature (e.g., problems with, they don’t care, holding me back, competing forces). Addressing these beliefs, through working with injured workers and relevant others, has the potential to improve the injured workers’ outlook. Efforts directed towards decreasing uncertainty, particularly as it relates to what might happen when the individual returns to the workplace, also appear to have the potential to facilitate a timely return to work. While in the current study beliefs tended to reflect difficulties, wants tended to be motivators. For those working to improve return-to-work outcomes, a strategy that focuses on highlighting the positives associated with returning to work appears to have the potential to prove fruitful. Other intervention opportunities center on helping people work through the tasks/processes they need to complete to move forward in the return-to-work process. A common hang-up point for the current participants was the search for the “right job.” While the desire to make choices that are in the worker’s best interest should be supported, those aiming to help people move through the process of returning to work should be aware that, although people often aspire to find new work after a serious injury or health condition, in most cases, returning to the pre-injury job is the final outcome (e.g., [28]). For people wanting to transition to a new job, a supportive strategy may be to highlight that it is generally easier to get a job when you have a job, and once back at work, there is always the option to pursue a job or career change. Additional intervention opportunities relate to improving access to resources, reducing waiting times, facilitating relationships, and addressing re-injury concerns.

### Strengths, limitations and methodological considerations

The current study had a number of strengths. Our participants had a variety of musculoskeletal conditions that included traumatic injuries, as well as episodic and chronic conditions. Our method enabled us to get a comprehensive capture of what people who are in the acute and sub-acute phases of work disability take into consideration when forming their expectations for returning to work. The focus group dynamic facilitated the exploration of the topic in free-flowing conversation. Participants were very forthcoming in sharing details of their injury, work experiences and personal lives. The analysis was performed with the aim of determining considerations that were more commonly referenced. Thus, study findings go some way towards providing the reader with a more generalized, and less idiosyncratic, understanding of what workers reference when forming their expectations for returning to work. In addition, so as to facilitate an understanding of the extent to which findings are applicable in other settings, we have provided contextual detail that can be used by the reader to evaluate the extent to which the conclusions drawn are transferable to other times, situations, and people.

Together with its strengths, the study has its limitations. While efforts were made to recruit persons with a range of return-to-work expectations, all study participants reported expecting to return to work. As such, only the factors that were considered by those who expect to return to work have been represented here. It is possible that people who do not expect to return to work might consider other factors when forming their expectations. While this is a limitation, research has shown that the vast majority of people do return to work following a work-disabling condition, with figures indicating that only just over 10% of workers’ compensation claimants are off work at 12 months following work-disability onset [29]. As such, our sample’s expectations are consistent with the return-to-work experience of the majority of the work-disabled population. A further limitation is that the participants in this study were all residents of Massachusetts and were working for employers based in the New England region of the United States at the time of their injury/onset of their work-disabling condition. In the US, there is some interstate variation in state-level welfare, unemployment, and Workers’ Compensation policies, which, in turn, may have influenced our participants’ considerations, particularly those of the economic context. Finally, the study was only of people off work due to a musculoskeletal condition. Influencing factors may be different for people with other conditions. With regards to gender balance, our sample contained more men than women at a rate of 3:1. We believe that this is a reflection of the selection criteria. Among those affected by a work-related injury, men are more commonly represented (ratio of approximately 2:1) [30]. At a general level, the rate of serious injury is much higher for men than women [31].

While our intention had been to use a group setting to gather the data, participant non-attendance resulted in the conduct of three two-on-one interviews. We found that this alternative format worked well, especially when we compared and contrasted the experiences of earlier participants. We did note some difference between the smaller and larger group sizes, with our sense being that group cohesion was better when the group was smaller. In the case of the larger group, we noted some group division and differing levels of participant engagement. With regards to our method of analysis, the strategy we employed was done so based on our aim to focus on influences that were more commonly mentioned. Future work will compare and contrast the influences that are referenced by persons who expect to return to work in the near future, with those of persons who do not expect to return until much later. The cross-tabulation of elements by lived-experiences was a technique that we learned about through attending a training course on the use of NVivo 10 (QSR International). Using this strategy allowed us to efficiently identify themes that were consistent with our less systematic observations, with the added benefit of facilitating easy retrieval of spoken content. Study findings provide insight for future research. A number of the identified themes suggest opportunities for modelling through the application of established constructs (e.g. fear avoidance, work identify, optimism and resilience). In addition, the current findings can be used to inform the development of a multi-dimensional measure of expectations for returning to work.

### Conclusion

People take many factors into consideration when forming their expectations for returning to work. These cover a range of personal and environment factors that can influence expectations in different ways. Considerations most frequently talked about involved: desires and feelings pertaining to themselves; feelings and beliefs together with access to resources as they related to their work context; and feelings concerning their disabling condition. Findings provide building blocks for further study and highlight opportunities for intervention aimed at improving return-to-work outcomes.
